# A novel UHPLC‒MS/MS method for quantitative analysis of zanubrutinib in rat plasma: application to an in vivo interaction study between zanubrutinib and triazole antifungal

**DOI:** 10.1186/s13065-023-01017-x

**Published:** 2023-08-30

**Authors:** Peng-fei Tang, Su-su Bao, Zhong-xiang Xiao, Wei-fei Xie, Xue-meng Wu, Hong-lei Ge, Chuan-feng Shao

**Affiliations:** 1 Affiliated Yueqing Hospital, Wenzhou Medical University, Wenzhou, Zhejiang, 325600 China; 2 Market Supervision Administration of Yueqing city, Wenzhou, Zhejiang, 325600 China

**Keywords:** Zanubrutinib, Drug–drug interaction, UHPLC–MS/MS, Pharmacokinetics

## Abstract

**Background:**

This study establishes a UHPLC‒MS/MS method for the detection of zanubrutinib and explores its interaction with fluconazole and isavuconazole in rats.

**Methods:**

A protein precipitation method using acetonitrile was used to prepare plasma samples using ibrutinib as an internal standard. Chromatographic separation and mass spectrometric detection of the analytes and internal standards were performed on a Shimadzu 8040 UHPLC‒MS/MS equipped with a Shim-pack velox C18 column (2.1 × 50 mm, 2.7 µm). Methanol and 0.1% formic acid-water were used as mobile phases. Intraday and interday precision and accuracy, extraction recoveries, and matrix effects of this method were determined. The linearity and sample stability of the method were assessed. Eighteen male Sprague‒Dawley (SD) rats were randomly divided into three groups with zanubrutinib (30 mg/kg) alone, zanubrutinib in combination with fluconazole (20 mg/kg) or zanubrutinib in combination with isavuconazole (20 mg/kg). Blood samples (200 µL) were collected at designated time points (ten evenly distributed time points within 12 h). The concentration of zanubrutinib was determined using the UHPLC‒MS/MS method developed in this study.

**Results:**

The typical fragment ions were m/z 472.15 → 290.00 for zanubrutinib and m/z 441.20 → 138.10 for ibrutinib (IS). The range of the standard curve was 1-1000 ng/mL with a regressive coefficient (R^2^) of 0.999. The recoveries and matrix effects were 91.9-98.2% and 97.5-106.3%, respectively, at different concentration levels. The values for intra- and interday RSD% were lower than 9.8% and 5.8%, respectively. The RSD% value was less than 10.3%, and the RE% value was less than ± 4.0% under different storage conditions. Analysis of pharmacokinetic results suggested that coadministration with isavuconazole or fluconazole significantly increased the area under the curve (1081.67 ± 43.81 vs. 1267.55 ± 79.35 vs. 1721.61 ± 219.36), peak plasma concentration (332.00 ± 52.79 vs. 396.05 ± 37.19 vs. 494.51 ± 130.68), and time to peak (1.83 ± 0.41 vs. 2.00 ± 0.00 vs. 2.17 ± 0.41) compared to zanubrutinib alone.

**Conclusion:**

This study provides information to understand the metabolism of zanubrutinib with concurrent use with isavuconazole or fluconazole, and further clinical trials are needed to validate the results in animals.

## Introduction

**Fig. 1:**
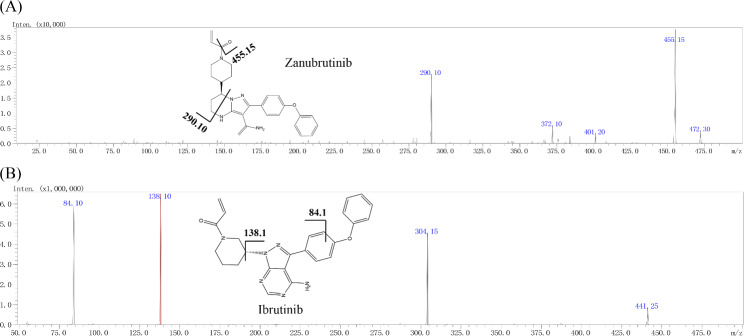
The chemical structures and mass spectra of zanubrutinib and IS in the present study

Zanubrutinib is a second-generation highly selective inhibitor targeting Bruton’s tyrosine kinase (Fig. 1) [1]. In 2019, zanubrutinib received accelerated approval by the U.S. Food and Drug Administration (FDA) for treating adult patients with recurrent or refractory lymphoma who received at least one kind of treatment [2]. It was further approved by the FDA for treating adult patients with Walden Strom’s macroglobulinemia and marginal zone lymphomas who received at least one prior anti-CD20 treatment in 2021. Zanubrutinib inhibits BTK activity by covalently binding to the 481st cysteine residue in the ATP binding pocket [3]. Research indicates that zanubrutinib shows almost 100% efficacy and that lasting BTK receptors inhibit effects in both peripheral blood mononuclear cells (PBMCs) and peripheral lymph nodes in patients with B-cell lymphoma [1]. Compared to the first-generation BTK inhibitor ibrutinib, zanubrutinib has shown similar potency at inhibiting BTK, but zanubrutinib was more than 20 times lower at inhibiting IL-2-induced kinase (ITK) [4]. Thus, zanubrutinib has lower off-target kinase inhibitory effects than ibrutinib, which contributes to reducing adverse reactions [4]. The pharmacokinetics results in patients with malignant lymphoma showed that zanubrutinib was absorbed rapidly and had good bioavailability after oral administration, and the time of peak concentration (T_max_).

was approximately 2 h [1]. After a single administration of 320 mg, the mean peak concentration (C_max_) of zanubrutinib was 658 ng/mL, and the mean half-life (t_1/2_) was approximately 4 h [1]. There is little accumulation in the body after repeated administration.

The results of in vitro experiments show that zanubrutinib is primarily metabolized to phenoxy phenyl hydroxylation metabolites through CYP3A4 [5]. A clinical study investigated the effect of itraconazole (CYP3A4 inhibitor) on the pharmacokinetics of zanubrutinib in healthy volunteers [6]. The results showed that when combined with itraconazole, the exposure of zanubrutinib in the body increased significantly, and Cmax and AUC increased to 154% and 143%, respectively. Invasive fungal infection (IFI) is a frequent complication in the treatment of lymphoma and is closely associated with the quality of life, therapy and prognosis of patients with lymphoma [7, 8]. Triazole antifungal drugs are the first choice in the treatment of malignant hematological diseases with invasive fungal infections [9–11]. Research shows that triazole antifungals such as fluconazole and isavuconazole are CYP3A4 enzyme inhibitors [12, 13]. Because the treatment of lymphoma patients with invasive fungal infection needs to address both antitumor and antifungal therapy, it is important to understand the drug–drug interaction (DDI) potential between zanubrutinib and triazole antifungal drugs. Hence, there is an urgent need to develop a fast and accurate technique to determine zanubrutinib concentrations in plasma. This will facilitate the discovery of potential drug interactions during combined administration via evaluation of pharmacokinetic parameters.

To date, only two articles have reported the determination of zanubrutinib concentrations in biological samples. Cheng et al. (2022) and Zhu et al. (2022) established quantitative methods for zanubrutinib in the plasma of mice and beagles, respectively [14, 15]. However, no article has reported a method for the determination of zanubrutinib concentration in rat plasma. In this study, a UHPLC‒MS/MS method was developed for the rapid quantitative determination of zanubrutinib in rat plasma. The commonly used parameters of specificity, selectivity, extraction recovery, matrix effect, precision, accuracy and stability were implemented to verify the feasibility and accuracy of the present method.

Finally, the interaction of different kinds of triazole antifungal drugs (fluconazole and isavuconazole) with zanubrutinib in rats was determined using the newly constructed methods.

## Materials and methods

### Chemicals and reagents

Ibrutinib (purity = 99.0%, internal standard, IS), zanubrutinib (purity > 99%), fluconazole (purity = 99.0%), and isavuconazole (purity = 99.0%) were obtained from Toronto Research Chemicals Inc. (Toronto, Ontario, Canada). The normal reagents for mass spectrometry and formic acid, including analytical-grade methanol and water, were obtained from Honeywell Burdick & Jackson (Muskegon, MI, USA) and J&K Scientific Ltd. (Shanghai, China).

### Animal experiments

The animal experiments were approved by the Animal Research Ethics Committee of Wenzhou Medical University (Ethics approval number: wydw 2021-0018). The animal-related procedures were in accordance with the Guide for Care and Use of Laboratory Animals of Wenzhou Medical University. Male Sprague–Dawley (SD) rats were purchased from the Experimental Animal Center of Wenzhou Medical University. Experimental animal production license number: SCXK 2021-0011.

The animals were raised in a suitable environment for 14 days before the experiment with appropriate temperature (25 ± 5 °C), humidity (50 ± 5%), light, and adequate food and water. Before the pharmacokinetics experiment, the animals were fasted for 12 h but had free access to drinking water.

### UHPLC–MS/MS detection method

An ultra-performance liquid chromatography system (Shimadzu Corp., Tokyo, Japan) was applied for chromatographic separation of the samples. The system contains an autosampler, an infusion pump, a vacuum degas unit, and a column oven.

The separation procedures were processed using a Shim-pack velox C18 column (2.1 × 50 mm, 2.7 μm) at 40 degrees Celsius. The mobile phases were 0.1% formic acid-water and methanol. The gradient elution mode (each injection = 3 min) was applied as follows: the first 0-0.5 min was maintained at 10% methanol, followed by linearly increasing to 80% methanol (0.5–0.8 min) and keeping 80% methanol for 0.8–1.5 min. Subsequently, it was linearly decreased to 10% methanol (1.5–2.5 min), followed by keeping 10% methanol for 0.5 min. At the stage of the analysis process, the sample injection volume and flow rate were set as 2 µL and 0.4 mL/min, respectively. As a result, both zanubrutinib (retention time 2.3 min) and ibrutinib (IS, retention time 1.9 min) exhibited high separation efficacies.

Triple quadrupole mass spectrometry (Shimadzu Corp., Tokyo, Japan) was utilized for sample detection. The parameters for this procedure were set as follows: the detector voltage, heating block temperature, flow of atomizing gas, and drying gas were set as 4.5 kV, 400 °C, 3 L/min and 5 L/min, respectively. Multiple reaction monitoring (MRM) mode was performed to detect the precursor and product ions of the IS and analyte. The first and second signal strength product ions were applied to quantitation and qualification, respectively, to ensure the specificity of the detection. In addition, the retention times of the IS and analyte were also considered to further improve the detection specificity. The MS data for IS and zanuubrutinib are listed in Table 1.

**Table 1:** MS parameters of zanubrutinib and ibrutinib

Analytes	Precursor Ion (m/z)	Product Ion 1 (m/z)	Collision Energy 1 (V)	Product Ion 2 (m/z)	Collision Energy 2 (V)
Zanubrutinib	472.15	290.00	40	455.15	36
Ibrutinib	441.20	138.20	31	84.20	45

### Calibration solution and QC sample preparation

A series of working solutions of zanubrutinib and IS in this study were diluted using methanol. Standard curve points and quality controls (QCs) for sample preparation were processed by the addition of the corresponding zanubrutinib working solution (10 µL) to blank rat plasma (90 µL). Finally, the concentrations for the standard curve points were defined as 1, 2, 5, 10, 20, 50, 100, 500 and 1000 ng/mL. The IS working solution was finally defined as 40 ng/mL, and quality control samples were defined as three concentrations (2, 40, 800 ng/mL). The solutions and samples were stored at -20 °C.

**Fig. 2:**
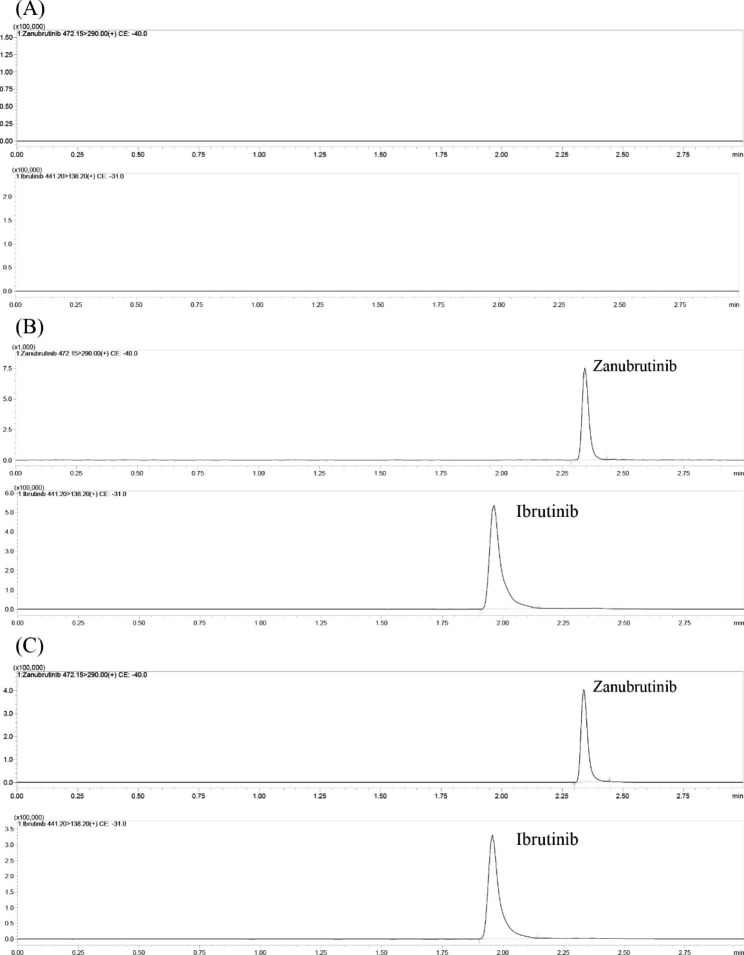
Representative UHPLC–MS/MS chromatograms of zanubrutinib and ibrutinib (IS). **(A)** blank plasma; **(B)** a blank plasma sample spiked with LLOQ concentration levels of zanubrutinib and IS; **(C)** a rat plasma sample obtained 1 h after oral administration of zanubrutinib. The plasma concentration of zanubrutinib was 230 ng/mL

### DDI study

Zanubrutinib, fluconazole and isavuconazole were dissolved in 0.5% carboxymethyl cellulose sodium (CMC-Na) solution. Eighteen SD rats were randomly split into three groups (Groups A-C). Group B and Group C were gavaged with fluconazole (20 mg/kg) and isavuconazole (20 mg/kg), respectively. The same dose (0.5% CMC-Na) was administered to Group A (control group). After half an hour, each rat was treated with 30 mg/kg zanubrutinib. Thereafter, rat blood samples (0.3 mL) from the caudal vein at a series of time points (0.083, 0.5, 1, 2, 3, 4, 5, 6, 8, and 12 h) were collected in heparin tubes. The blood samples were centrifuged at 13,000 × g for 10 min at 4 °C, followed by separation of plasma and storage at -80 °C.

### Euthanasia

Euthanasia of experimental animals was performed using the anesthesia method according to the AVMA Guidelines for the Euthanasia of Animals. After completion of the experiment, all experimental animals were euthanized by intravenous pentobarbital (150 mg/kg). After ensuring that the animals were free of life pointers, they were packaged and cremated.

**Table 2:** Recovery and matrix effect of zanubrutinib in rat plasma (n = 6)

Analyte	Concentration (ng/mL)	Recovery (%)		Matrix effect (%)	
		Mean ± SD	RSD(%)	Mean ± SD	RSD(%)
Zanubrutinib	2	92.0 ± 10.2	11.1	104.4 ± 9.9	9.5
	40	98.2 ± 11.4	11.6	97.5 ± 6.0	6.1
	800	97.0 ± 5.8	6.0	106.3 ± 6.6	6.2

### Sample preparation

Prior to the experiment, the − 80 °C stored rat plasma samples were placed at room temperature for thawing. At the protein precipitation stage, 40 µL IS, 200 µL acetonitrile, and 100 µL plasma were collectively added to a 1.5 mL centrifuge tube, vortexed to mix thoroughly, and centrifuged at 12,000 × g for 10 min. Afterwards, 100 µL supernatant was transferred to a centrifuge tube and gently mixed with 100 µL ultrapure water for UHPLC–MS/MS analysis.

### Validation of methods

Prior to using our method for testing, we first validated the parameters (stability, selectivity, recovery, etc.) in accordance with FDA method validation guidance [16].

**Table 3:** Precision and accuracy for zanubrutinib of QC samples in rat plasma (n = 6)

Analyte	Concentration (ng/mL)	Intraday			Interday		
		Mean ± SD	RSD(%)	RE(%)	Mean ± SD	RSD(%)	RE(%)
Zanubrutinib	1	0.97 ± 0.10	9.8	-2.7	1.00 ± 0.06	5.8	-0.2
	2	2.04 ± 0.11	5.2	2.2	2.02 ± 0.03	1.4	1.1
	40	40.29 ± 1.25	3.1	0.7	40.87 ± 0.62	1.5	2.2
	800	786.41 ± 68.89	8.8	-1.7	803.16 ± 15.34	1.9	0.4

#### Selectivity and specificity

The selectivity of the method refers to the ability to accurately determine the target compound from a biological sample. Herein, the selectivity of the method was assessed by using the method to determine blank plasma from six different rats, blank plasma containing the target compound, and plasma collected from rats after oral administration of the target drug.

#### Linearity and LLOQ

Eight various concentrations (1-1000 ng/mL) of standard samples were applied to establish the standard curves. The experiments were performed on three different days. The degree of linearity could be estimated using R-squared (peak area ratios versus concentrations). The lower limit of quantification (LLOQ) is defined as the lowest amount of zanubrutinib that can be quantitatively determined under acceptable accuracy and precision. A signal-to-noise ratio of at least 10:1 was applied to estimate the LLOQ.

**Table 4:** Summary of the stability of zanubrutinib in rat plasma under different storage conditions (n = 6)

Analyte	Concentration (ng/mL)	Room temperature		4 °C		Three freeze‒thaw		-80 °C	
		RE(%)	RSD(%)	RE(%)	RSD(%)	RE(%)	RSD(%)	RE(%)	RSD(%)
Zanubrutinib	2	-0.2	6.1	1.3	5.0	-1.8	4.1	2.5	5.2
	40	-1.4	4.1	1.4	7.1	2.6	4.0	1.5	10.3
	800	-2.3	5.7	1.0	5.1	-0.4	6.5	4.0	4.7

#### Recovery and matrix effect

Blank plasma from 6 different rats and 3 different concentrations of zanubrutinib QC standards (2, 40 and 800 ng/mL) were utilized to evaluate the recovery and matrix effects. The recovery was assessed by comparison of the peak area ratios of the QC sample and the extract blank plasma containing equal amounts of the test compound. The matrix effect was determined by comparison of the peak area ratios of the extract blank plasma containing a definite concentration of the test compound and standard solution of the same concentration.

#### Accuracy and precision

Three different concentrations of rat plasma QC samples were evaluated on three different days. To measure the accuracy and precision of our method, the parameters of relative error (RE%) and relative standard deviation (RSD%) were applied to determine whether the accuracy and precision conformed to the specification (whether they were within 15%).

#### Stability

Under a series of storage conditions, each concentration of rat plasma QC samples (2, 40 and 800 ng/mL) was evaluated in 6 replicates to explore the stability of the method. This experiment determines the stabilities of the QC samples during analyses and different storage conditions. This experiment contained stability during analysis (4 h at room temperature), cycles for freezing-thawing (three times), storage for short-term storage (4 °C for 24 h), and storage for long-term storage (-20 °C for a month). The testing results with RSD% (< 15%) and RE% (± 15%) were considered stable.

**Fig. 3:**
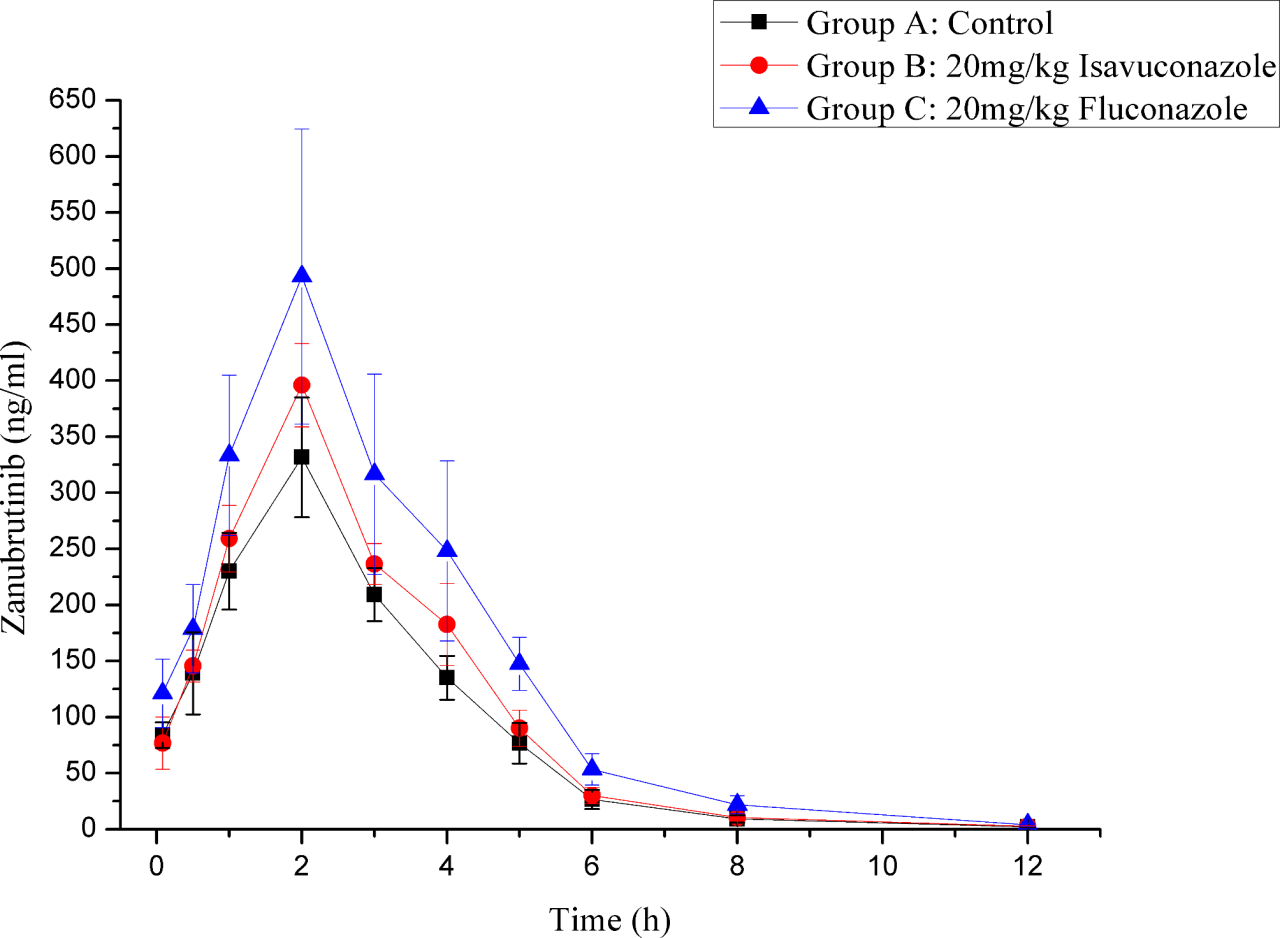
Mean plasma concentration-time curve of zanubrutinib in different treatment groups of rats. Group A: the control group (0.5% CMC-Na), Group B: 20 mg/kg isavuconazole and Group C: 20 mg/kg fluconazole (n = 6, mean ± SD).

## Results and discussion

### Development and optimization of the Method

#### Optimization of chromatographic conditions

The mode for elution, the composition of the mobile phase, the temperature of the column and the types of columns for separation were optimized to accomplish more efficient separation of zanubrutinib and IS. The optimized method possessed higher specificity and sensitivity, better peak shapes and a shorter running time. Herein, a series of columns, including the variation of column lengths, packing materials and particle sizes, were evaluated. The Shim-pack velox C18 (2.1 × 50 mm, 2.7 μm) column exhibited optimal separation, retained value, and chromatographic peak form. In addition, different types of mobile phase compositions were assessed, including methanol, acetonitrile, water with/without 0.1% formic acid, etc. As a result, methanol and 0.1% formic acid-water formed the mobile phase, which showed an optimal peak shape and separation effect. Moreover, flow rates of 0.3 to 0.5 mL/min, column temperatures (20–40 °C), and isocratic and gradient elution were also assessed. By comparison, the run time of the optimal method was 3 min, and the retention times for zanubrutinib and IS were 2.34 and 1.98 min, respectively. The parameters for the optimal method were as follows: mobile phase (methanol and 0.1% formic acid-water), gradient elution (flow rate 0.4 mL/min), and column temperature (40 °C). Initially, the mobile phase ratio of methanol and 0.1% formic acid-water was set as 1:9 (v/v) for elution. Afterwards, the methanol volume percentage gradually increased to 80% at 0.5 min and remained at 80% until 2 min. Thereafter, the volume percentage of methanol gradually decreased to 10% until the elution program finished at 3 min. The characteristic chromatograms of the blank control, blank rat plasma supplemented with zanubrutinib and IS standard, and rat plasma after administration are plotted in Fig. 2. It can be seen from the chromatogram that the peaks obtained were sharp, symmetrical and interference-free, indicating that the assay has good specificity and does not suffer from the interference of endogenous substances and commonly used chemicals. The time of each assay is 3 min, which can meet the requirements of high-throughput detection.

**Table 5:** The main pharmacokinetic parameters of zanubrutinib in different treatment groups of rats

Parameters	Unit	Group A	Group B	Group C
AUC_(0−t)_	µg/L*h	1081.67 ± 43.81	1,267.55 ± 79.35*	1721.61 ± 219.36**
AUC_(0−∞)_	µg/L*h	1085.49 ± 44.17	1272.62 ± 77.99*	1729.93 ± 218.06**
MRT_(0−t)_	h	2.71 ± 0.10	2.76 ± 0.07	2.95 ± 0.14*
MRT_(0−∞)_	h	2.76 ± 0.09	2.82 ± 0.09	3.00 ± 0.14*
t_1/2_	h	1.34 ± 0.11	1.42 ± 0.56	1.37 ± 0.16
T_max_	h	1.83 ± 0.41	2.00 ± 0.00*	2.17 ± 0.41*
CLz/F	L/h/kg	27.67 ± 1.09	23.64 ± 1.35*	17.59 ± 2.37**
Vz/F	L/kg	53.25 ± 3.64	48.95 ± 21.13	34.80 ± 6.57**
C_max_	ng/mL	332.00 ± 52.79	396.05 ± 37.19*	494.51 ± 130.68**

Group A: the control group (0.5% CMC-Na), Group B: 20 mg/kg isavuconazole and Group C: 20 mg/kg fluconazole. (n = 6, mean ± SD). Compared with Group A, *P < 0.05, **P < 0.01

#### Optimization of mass spectrometer conditions

To better detect zanubrutinib and IS, the parameters for the mass spectrometer, including the drying gas flow rate, atomizing gas, detection modes, and collision energy (CE), were evaluated. The optimized parameters for the drying gas flow rate and atomizing gas were 15 and 3 L/min, respectively. The gas pressure of CID and temperature of the desolvation line (DL) were set as 17 kPa and 250 °C, respectively. The CE values for positive ion mode are displayed in Table 1.

#### Optimization of sample preparation and IS

The protein precipitation method and solvent extraction are the most widely used methods for extracting compounds from biological samples. Compared with solvent extraction, acetonitrile-based protein precipitation was more suitable for zanubrutinib and IS with high recovery (91.9-98.2%) and stable chemical properties in this study. Therefore, the acetonitrile precipitation method was applied to pretreat the samples.

To select suitable ISs, the commonly used ISs dextromethorphan and nifedipine and similar ISs gefitinib and ibrutinib were assessed. Our results indicated that ibrutinib possessed remarkable sensitivity and stability. In addition, ibrutinib exhibited similar chemistry and retention time compared to zanubrutinib. Furthermore, the positive ion monitoring mode was suitable for both ibrutinib and zanubrutinib.

### Method validation

#### Selectivity and specificity

As plotted in Fig. 2, the relative retention times for zanubrutinib and IS are 2.34 min and 1.98 min, respectively. Our method did not interfere with commonly used chemicals or endogenous substances.

#### Linearity and LLOQ

The relative peak area (zanubrutinib/IS) and its corresponding serum concentration were applied to linear regression analysis based on the least squares method. The results showed that the linear relationships were in good agreement with the concentration range of 1-1000 ng/mL with a regressive coefficient (R^2^) of 0.999. The LLOQ for zanubrutinib was 1 ng/mL, and the corresponding RSD% and RE% of our method were < 9.8% and within 2.7%, respectively.

#### Recovery and matrix effects

The recovery and matrix effects (MEs) for the extraction of the zanubrutinib QC samples from low to high concentrations are listed in Table 2. The average recoveries of zanubrutinib at concentrations of 2, 40 and 800 ng/mL were 91.9%, 98.2%, 97.0%, respectively, and the MEs were 104.4%, 97.5%, 106.3%, respectively. Our results indicated that the proposed method can ignore the effect of MEs in daily detection and has the advantage of high recovery.

#### Accuracy and precision

To assess the accuracy and precision of our method, the RE% and RSD% were calculated for QC samples of various concentrations, including LLOQ (Table 3). Our results showed excellent stability and reproducibility in that the values for intra- and interday RSD% were lower than 9.8% and 5.8%, respectively. The RE% values were in the ranges of -2.7–2.2% and − 0.2–2.2%.

#### Stability

Calculation of the RE% and RSD% values under 4 different storage conditions (room temperature, 4 degrees Celsius refrigeration, freeze thawing, cryopreservation for long-term) for QC samples was applied to assess the short/long-term stability of the analytes. As shown in Table 4, the stability results for zanubrutinib showed that the RSD% value was less than 10.3%, and the RE% value was less than ± 4.0%. According to the above results, we can conclude that zanubrutinib in plasma was stable under the assessed storage conditions.

### DDI Study

The previously constructed UHPLC‒MS/MS method was successfully applied to research the drug‒drug interactions between zanubrutinib and two kinds of triazole antifungal drugs in rats.

As shown in Fig. 3; Table 5, when zanubrutinib was concomitantly used with isavuconazole, the AUC_(0-t)_, AUC _(0-∞)_, T_max_ and C_max_ of zanubrutinib were markedly increased (p < 0.05), while CLz/F was decreased significantly (p < 0.05) compared to the control group. These results demonstrated that isavuconazole has an obvious inhibitory effect on the metabolism of zanubrutinib, resulting in a remarkable increase in total systemic exposure to zanubrutinib. For zanubrutinib in combination with fluconazole, the increasing amplitude of AUC_(0-t)_, AUC_(0-∞)_, T_max_ and C_max_ of zanubrutinib was greater than that in the B group. The above results suggested that fluconazole has a stronger inhibitory effect on zanubrutinib metabolism than isavuconazole. Therefore, it should be noted that patients are more vulnerable to severe adverse reactions when zanubrutinib and fluconazole/isavuconazole are coadministered due to the elevated plasma levels of zanubrutinib. If coadministration of the above drugs is inevitable, it is better to reduce the dose of zanubrutinib. As the interaction study of zanubrutinib and two kinds of triazole antifungal drugs was performed in a limited number of rats, the results still need to be verified in further clinical trials.

## Conclusion

The present study constructed an ultra-performance liquid chromatography-tandem mass spectrometry method to detect zanubrutinib in rat plasma with high efficiency and accuracy. As an example, we successfully applied this method to the drug interaction study of zanubrutinib and two kinds of triazole antifungal drugs (isavuconazole and fluconazole) in rats. Fluconazole possessed a stronger inhibitory effect than isavuconazole on the metabolism of zanubrutinib in rats, and both increased total systemic exposure to zanubrutinib. In view of the complexity of cancer patients in the real world, further human trials are urgently needed to validate the results of animal experiments.

## Data Availability

All data and material analyzed or generated during this investigation are included in this published article.
